# Official Interview with Prof. Dr Max Hilz | 23^rd^ AMN Congress

**DOI:** 10.25122/jml-2026-1015

**Published:** 2026-08

**Authors:** Stefana-Andrada Dobran, Alexandra Gherman

**Affiliations:** 1 RoNeuro Institute for Neurological Research and Diagnostic, Cluj-Napoca, Romania

**Interviewee: Prof. Dr Max Hilz**

**Interviewer: Ms. Ștefana-Andrada Dobran**

Prof. Dr. med. Dr. med. habil. Dr. h.c. Max J. Hilz, M.D., FEAN, FAAN is an internationally renowned neurologist specializing in clinical neurophysiology, neurological intensive care medicine, and disorders of the autonomic nervous system (ANS).

Throughout his academic career, Prof. Hilz has held prominent positions at leading institutions in Europe and the United States. He was Professor of Neurology, Medicine, and Psychiatry at New York University, Chair of Autonomic Neurology at the Institute of Neurology, Queen Square, London, and Professor of Neurology at the University of Erlangen-Nuremberg, Germany. Since 2015, he has also served as Adjunct Professor of Neurology at the Icahn School of Medicine at Mount Sinai, New York.

Prof. Hilz has played major leadership roles in international neurological organizations. He has served as Chair of the Autonomic Disorders Research Group of the World Federation of Neurology, former Chair of the Autonomic Nervous System Scientific Panel of the European Academy of Neurology, and former Chair of the Autonomic Section of the American Academy of Neurology. He has also served as former Chair of the German Autonomic Society and Past President of the European Federation of Autonomic Societies.

His scientific expertise is further reflected in his editorial activities, including roles with the Journal of the Neurological Sciences, Clinical Autonomic Research, Neurology and Therapy, European Journal of Neurology, and Autonomic Neuroscience: Basic and Clinical. He has also contributed to international clinical guidelines and has served as an advisor to the European Medicines Agency on issues related to the autonomic nervous system, has co-authored several guidelines (e.g., on syncope, erectile dysfunction, orthostatic hypotension, supine hypertension, and diabetic neuropathy), and he published 370 original and review articles and book chapters, as well as the textbook Autonomic Disorders in Clinical Practice.


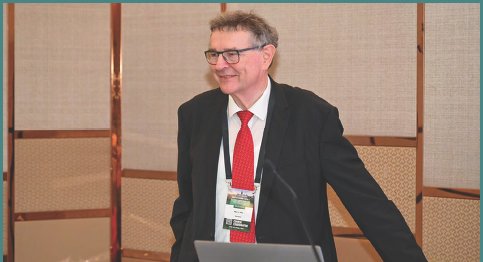


**S.D.: Dear Professor Max Hilz, this year you are coordinating the** **Hands-on Teaching Course on Acute and Chronic Stress, Sequelae, and Ways of Relaxation with Focus on Autogenic Training****, as part of the 23^rd^ Congress of the Academy for Multidisciplinary Neurotraumatology (AMN). What inspired the focus on stress and autonomic regulation within the context of neurotrauma and neurological recovery?**

**M.H.:** That is a very easy question to answer. We are all stressed. I barely know anyone in the medical business who does not experience stress. But, of course, our patients are also stressed.

We conducted studies that evaluated the so-called autonomic nervous function–the interplay between the sympathetic system and the parasympathetic system–in patients who had experienced a traumatic brain injury six months or even two or three years previously, and we were able to show that there is an imbalance between these two functions.

As you know, for lay people, the sympathetic system is the one that is very active when we have to run up the stairs, when we have to run away from danger, or when we are confronted with a threatening situation, while the parasympathetic system slows us down. It is most active during deep sleep stages, and it is often referred to as the “rest and digest” system.

When you look at patients who had a traumatic brain injury, a stroke, or any other central nervous system dysfunction, in most cases, particularly in traumatic brain injury, you find there is relatively too much sympathetic activity under resting conditions and not enough parasympathetic activity. In other words, one of the key components of stress is too much sympathetic and too little parasympathetic activation – that is exactly what you see in patients who had previously experienced a traumatic brain injury.

The closer [you are] to the injury–I assume, we have not studied that, but it is very reasonable to assume that–the more stress, the more sympathetic activity you have and the less parasympathetic activity. During the acute phase of traumatic brain injury, there are even sympathetic hyperactivity crises, or ***sympathetic storms***, which can be life-threatening.

Of course, immediately after a severe traumatic brain injury, it is difficult to teach the person to relax, because they might not even be conscious. But afterwards, relaxation becomes very important. If the autonomic system remains out of balance, there is a high risk of episodes of excessively high sympathetic activity, which are dangerous. They compromise cardiovascular function and can lead to myocardial infarction or cardiac arrhythmia.

Many of these problems can be avoided if people learn to calm down, to relax. One of the many options for doing this is ***autogenic training*.**

Did the people who invent the autogenic training understand all those mechanisms? Probably not, because in those days, very little was known about the autonomic nervous system. Actually, the man who invented it spoke about the vegetative nervous system, which is the same system. The first textbooks of experts in the field were published in 1920 by Ludwig Robert Müller, a German internist, followed in 1921 by the famous textbook by Langley, which is known worldwide, as it is in English. However, the book about the autonomic nervous system was first released in 1927. So, I doubt that they were familiar with all the processes that happen when you learn to relax, particularly during autonomic training.


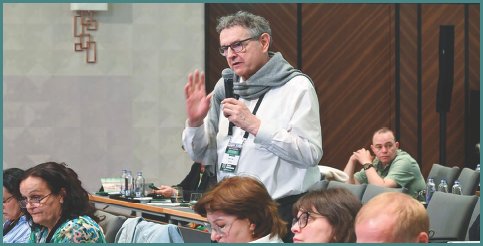


Today, however, studies clearly demonstrate these effects. Why is there an urgent need for people who work with patients who have experienced a traumatic brain injury to teach themselves how to relax? Because the medical staff, the nurses, the physiotherapists, the social workers, and, of course, the physicians, are also under stress. Nonetheless, the important part is to teach the patient how to mitigate the stress that is kind of post-traumatic and then is part of their daily life. They have to learn how to cope with it.

**S.D.: How often do these episodes, these sympathetic storms, appear after a trauma?**

**M.H.:** Well, how often it appears immediately after a trauma depends, again, on the type of traumatic brain injury. If it is moderate to severe traumatic brain injury, the risk is far higher than in a mild traumatic brain injury. I cannot give you a precise number, but if it appears, it is a very complicated situation, very difficult to manage, and it can have life-threatening consequences.

So, in the aftermath, the further you are from the moment when the trauma occurred, [the more the situation changes]. When the patients we tested went back to their initial work—regardless of whether they had a mild, moderate, or severe brain injury— their risk of having the so-called sympathetic crisis or storm that occurs in intensive care units is, I think, is rather limited. None of our patients showed abnormal clinical parameters, such as blood pressure, heart rate, body temperature, or respiratory frequency. Everything appeared normal, and they did not realize that their autonomic control was different.

The human body is remarkably capable of adapting. Many people live with high blood pressure, reflecting chronically increased sympathetic tone, and continue to function without realizing it. Is that healthy? Definitely not. High blood pressure increases the risk of stroke, myocardial infarction, and many other complications. If we monitor these patients–which we should–then we see, “*Aha, the balance between sympathetic and parasympathetic activity is out of range*”. They probably do not even know that, but it is not healthy, it is not good for them. They should bring it into balance in order to be able to manage really dangerous, stressful situations where they have to be able to work with both branches of their autonomic nervous system.

**S.D.: The course combines scientific understanding with practical approaches, such as autogenic training. What do you hope that the participants will take from this experience, both professionally and personally?**

**M.H.:** It is evident that the people who come to the course are the ones who are interested in learning how to relax, because they feel that they are constantly exposed to stress. We all adapt to stress, and we master stress for a long time, but sooner or later the battery runs empty and then it is often too late to be able to really cope with dangerous situations. However, I think it would be very good if people realize that—there are many people who say, “*I don't believe in this, it's not going to work with me*.” — if you want to relax, you can relax.

I know prominent neurologists who told me “*I'm very stressed, I'm constantly stressed, and I tried everything, I tried yoga and other types of techniques, it's not going to work*.” And I showed them that if you want to relax, it works. If they want to prove to me that it does not work, it is not going to work. If you say, “*No, I won't relax*,” then do not waste your time and don't waste mine. But anyone who is really interested and who thinks “*I will benefit, and I want to see whether I can relax myself*,” will benefit—that is a take-home message. Try to continue learning it, getting deeper into it, and you will very quickly advance to a degree where it takes very little to relax.

Do you like to go to the dentist?

**S.D.: Absolutely not**.

**M.H.:** Why? Because you are afraid of it. It is a stressful situation.


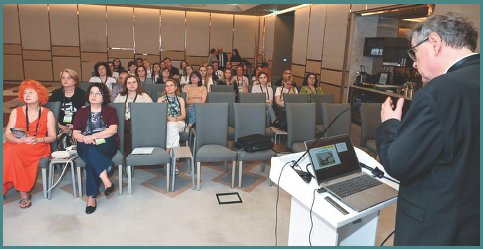



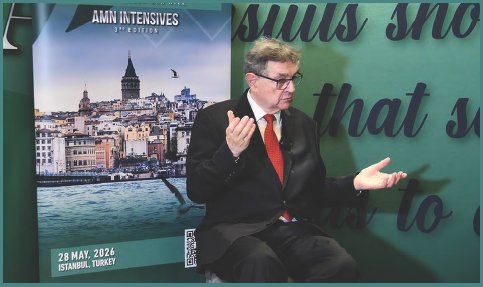


Do you like the sound of the drill? No. Some people hear a drill, and it reminds them “*Oh, that doesn't sound good. I already feel the pain*.” even when there is nothing happening. With autogenic training you can relax during a visit to the dentist, and he drills, and you relax totally.

I participated in many research studies as a volunteer. I once had to participate in a gastroscopy, and they even took biopsies. That was during a regular working day, and I said, “*They need someone in my age group, so I have to volunteer*.” The guy said, “*Give me your arm and we will inject you with a little bit of sedation*.” I said “*Are you out of your mind? I have to work*.” But I applied the relaxation technique, and I swallowed this big device that they put down your esophagus and into the stomach. That is not at all a problem if you know how to relax.

This is a stressful situation because you are afraid of it, but there is nothing to be afraid of. Much of what we are afraid of is in our head. We think going to the dentist is painful. Not necessarily. That is a practical example of how you can avoid stress. I could give you a thousand other examples, but we are running out of time.

In daily practice, there are a lot of stressful situations. Another emergency patient comes in and you already have two to handle. You still must be focused. You must master the difficult challenge of this stressful situation with patient number one, two, and now even three. And you still must have a clear mind when you are stressed; you do not necessarily make the right decisions. It helps to be able to relax, and if you learn the benefits, you also realize, “*this should be very good for my patients*.”

Once participants experience these benefits themselves, they will realize that the same techniques could also benefit their patients.

**S.D.: So, you hope that through this course you will encourage the participants to share the techniques**.

**M.H.:** Well, of course, otherwise it would be a waste of time.

I know some colleagues, even some prestigious ones, who say, “*I don't believe in this*.” There is nothing to believe. This is not a philosophy. This is not something where some mystic dogmas are implemented. *This is physiology, basic physiology*.

We learn very little about the autonomic nervous system in medical school, but anyone who wants to understand how it works should come to the course. I will explain every step before we do a 30–40-minute training session, and people will understand that this is really based on physiology.

*If I am healthy, I will be able to relax. Very easy*.

**S.D.: At the personal level, what would you envision as an add-on within your collaboration with the AMN?**

**M.H.:** This is the first time that the AMN has invited me to teach a seminar where I first talk about acute and chronic stress and its consequences, which are abundant—from having a syncope to dying and everything in between. I will then talk about different techniques which allow people to cope with stress. I believe you should teach what you know best. Since I have been taught autogenic training more than 40 years ago, I think that is what I must carry on and pass this knowledge on to others.

I would be happy to show as many of our colleagues as possible that this is something that will benefit them, but also their patients. And for whom are we working? Not for our wallet, but for our patients. It is something that, particularly in patients who have neurotrauma, can be extremely helpful. It is not a cure for neurotrauma, but it is a very important adjunct therapy that improves their quality of life and prognosis.

I would envision that if the AMN feels that this is a useful technique—I know it is, but the people who make the decision must be convinced—it would be helpful to teach more and more colleagues about this technique and to teach them how to relax themselves. Once they feel that, they will say, “*Okay, I can do it. Why not my patients?*” And that would be, of course, a very good outcome of this adventure.

